# Schlieren imaging and video classification of alphabet pronunciations: exploiting phonetic flows for speech recognition and speech therapy

**DOI:** 10.1186/s42492-024-00163-w

**Published:** 2024-05-22

**Authors:** Mohamed Talaat, Kian Barari, Xiuhua April Si, Jinxiang Xi

**Affiliations:** 1 Department of Biomedical Engineering, University of Massachusetts, Lowell, MA 01854 USA; 2 Department of Aerospace, Industrial, and Mechanical Engineering, California Baptist University, Riverside, CA 92504 USA

**Keywords:** Alphabet pronunciation, Speech flows, Articulatory phonetics, Video classification, Schlieren, Long short-term memory, Sequential learning

## Abstract

Speech is a highly coordinated process that requires precise control over vocal tract morphology/motion to produce intelligible sounds while simultaneously generating unique exhaled flow patterns. The schlieren imaging technique visualizes airflows with subtle density variations. It is hypothesized that speech flows captured by schlieren, when analyzed using a hybrid of convolutional neural network (CNN) and long short-term memory (LSTM) network, can recognize alphabet pronunciations, thus facilitating automatic speech recognition and speech disorder therapy. This study evaluates the feasibility of using a CNN-based video classification network to differentiate speech flows corresponding to the first four alphabets: /A/, /B/, /C/, and /D/. A schlieren optical system was developed, and the speech flows of alphabet pronunciations were recorded for two participants at an acquisition rate of 60 frames per second. A total of 640 video clips, each lasting 1 s, were utilized to train and test a hybrid CNN-LSTM network. Acoustic analyses of the recorded sounds were conducted to understand the phonetic differences among the four alphabets. The hybrid CNN-LSTM network was trained separately on four datasets of varying sizes (i.e., 20, 30, 40, 50 videos per alphabet), all achieving over 95% accuracy in classifying videos of the same participant. However, the network’s performance declined when tested on speech flows from a different participant, with accuracy dropping to around 44%, indicating significant inter-participant variability in alphabet pronunciation. Retraining the network with videos from both participants improved accuracy to 93% on the second participant. Analysis of misclassified videos indicated that factors such as low video quality and disproportional head size affected accuracy. These results highlight the potential of CNN-assisted speech recognition and speech therapy using articulation flows, although challenges remain in expanding the alphabet set and participant cohort.

**Supplementary Information:**

The online version contains supplementary material available at 10.1186/s42492-024-00163-w.

## Introduction

Speech is an intricately orchestrated activity that requires precise management of the vocal tract’s shape and movements to produce clear and understandable sounds [1]. Articulation therapy focuses on improving an individual’s ability to produce clear and correct speech sounds [2]. This therapy is commonly used for children and adults who have difficulty pronouncing certain sounds or words, which affects their overall speech clarity and communication effectiveness. The initial step typically involves the speech-language pathologist listening to the patient speaking in various contexts to identify the problematic sounds and understand the underlying causes [3]. One commonly used procedure at this stage is the oral-motor assessment, which examines the physical capabilities of the mouth, including the strength, coordination, and movement of the lips, jaw, and tongue. Successful articulation therapy results in clearer speech, significantly enhancing communication effectiveness and boosting confidence in social, educational, or professional settings.

Patients with hearing and speech disorders face more challenges than those with only speech disorders in following speech therapy due to their lack or weak feedback from the ear [4]. The brain requires multiple forms of feedback to iteratively correct the oral motors for accurate pronunciation. Other techniques are often implemented to help patients generate more meaningful feedback, such as placing a fingertip on the lips or cheek to feel motion or exhaling onto a piece of paper to feel the flow [5]. While these methods are easy to implement and have proven useful in helping patients correct their articulations, they are limited by indirect feedback and interference with normal speech. Thus, a new method that provides direct and undisruptive sensory feedback to patients with hearing disorders is desirable to improve therapy outcomes and shorten therapy duration [6].

The exhaled speech flows are thought to be closely related to oral motor control rather than the acoustics traditionally used for speech diagnosis [7–10]. An articulated sound results from the integration of the vocal source (phonation) and vocal tract (resonance), encompassing vibroacoustic (vocal fold collisions and structure vibrations), aeroacoustics (boundary layer vortices, main flow turbulences), and frequency-wise pressure-structure interactions (attenuation and amplification) [11]. The first primarily involves humming sounds, the second frequently includes noise, while the third leads to intelligible articulation unique to the speaker. The expiratory speech flow directly stems from the instantaneous oral-motor configuration (the resonator at that moment) shaped by the lips, tongue, teeth, oral cavity, and velum [12]. Therefore, any changes to this configuration will result in a distinct flow topology [13, 14]. Conversely, an altered flow topology could potentially be traced back to the structural anomaly [15]. It is theorized that by examining expiratory speech flows from patients with speech disorders, it may be possible to identify underlying anatomical or physiological factors, enabling patient-specific interventions/therapies. Similarly, speech flows could provide real-time feedback on the outcomes of these interventions/therapies.

A schlieren optical system facilitates the visualization of airflows through light refraction caused by heterogeneous air densities [16, 17]. This technique has often been applied to visualize shock waves [18, 19], ultrasonic standing waves [20], respiratory flows [21–23], liquid flows [24], and turbulence [25]. Schlieren imaging has been used in a limited number of studies focused on speech production. Tomaschek et al. [26] recorded the flow dynamics from lip, nasal, and vocal speeches using a schlieren system and quantitatively compared the flow intensity nasal and non-nasal sounds, revealing that delayed uvular closure nasalized the vowel sound. Furthermore, the flow variations following lip closure, as described in other studies [27, 28]. Lorenc et al. [29] used an acoustic camera approach to investigate Polish nasalized vowels and suggested that the acoustic pressure distribution was dependent on the resonance location (i.e., nasal, oral, velopharyngeal, etc.). The sequence and percentage share of oral and nasal resonances were also determined from the acoustic field of Polish nasalized vowels. Rowell et al. [30] compared speech airflows of nasal and oral vowels in French using the schlieren imaging technique and noted higher flow intensities for nasal vowels than oral vowels, as well as notable inter-speaker variability in flow patterns. Challenges associated with environmental factors for recording speech flows were also highlighted in as study [30]. Harvey et al. [31] studied acoustic waves propagating in the air using the high-speed schlieren technique and demonstrated that audio signals could be inversely recovered from schlieren-recorded videos, termed “schlieren microphone.”

Video classification has progressed significantly with the advent of deep learning, facilitating the integration of a pre-trained convolutional neural network (CNN) with a long short-term memory (LSTM) network. In this integrative approach, video frames are initially transformed into feature vectors by the convolutional network, capturing essential attributes of each frame [32, 33]. These vectors are subsequently fed into an LSTM network, which captures temporal information inherent in the video frame sequences [34]. The final architecture merges layers from both the convolutional and LSTM networks, enabling video label classification. This approach ensures that the classifier accounts for both the spatial characteristics derived from individual frames and the temporal continuity inherent in the video, allowing for a comprehensive video classification strategy. The hybrid CNN-LSTM method has seen increasing application in fields such as online video categorization [35], behavior/activity recognition [36], natural language processing [37], weather broadcasting [38], auto-driving [39], lung sound diagnosis [40], and detecting wake-sleep patterns [41].

This study aimed to evaluate the feasibility of using video classification to distinguish phonetic alphabet pronunciations (i.e., /A/, /B/, /C/, and /D/) captured using the schlieren imaging technique. It is hypothesized that the pronunciation of each letter will produce unique spatiotemporal flow patterns that differentiate it from other letters, and that certain features will remain consistent across participants, despite the presence of significant inter-participant differences. The focus of this study is on the video classification of the first four alphabetic letters. Specific aims include:

1. Develop a schlieren system and check the quality of recorded speech flows.
2. Develop a dataset of 640 videos recording articulations of /A/, /B/, /C/, and /D/.
3. Find the minimal number of videos required to train an accurate network.
4. Evaluate the performance of a trained network on speech videos from other participants.
5. Evaluate the effect of continuous training on classification performance.

## Methods

### Study design

The first four letters of the English alphabet, /A/, /B/, /C/, and /D/, were selected to assess the feasibility of artificial intelligence (AI)-based differentiation of speech flows acquired using schlieren photography. This selection was based on their notable differences and similarities, offering various levels of difficulty for classification. For example, /A/ is a vowel pronounced with no stricture in the vocal tract, while /B/, /C/, and /D/ are consonants articulated with complete or partial closure of the vocal tract [42]. Among these consonants, the phonation location varies: /B/ is articulated at the lips with a closing-opening motion, /C/ is produced at the closed teeth by forcing air through the teeth crevice, and /D/ is generated at the tongue tip that presses against the alveolar ridge. Notably, external observations of /A/ and /D/ pronunciations show minimal differences in lip and jaw movements, underscoring the necessity for additional biomarkers to disclose hidden tongue movements for more accurate classification.

Speech flows from two participants (i.e., first participant (P1) and second participant (P2)) were recorded using a schlieren optical system at 60 frames per second. The study received approval from the institutional review board at UMass Lowell, and both participants provided written consent. Audio recordings of the alphabet pronunciations were made simultaneously with the video recordings. These videos were then segmented into individual clips of one second each, with the highest expiratory flow rates typically occurring around the midpoint of each clip.

To create the classification database, a minimum of eighty separate video clips per letter and per participant were prepared, resulting in a total of more than 640 (80 × 4 × 2) video clips. To determine the minimum number of training videos required for an accurate network (i.e., > 90% accuracy), the CNN-LSTM model was trained separately on four datasets, containing 20 × 4, 30 × 4, 40 × 4, and 50 × 4 videos, respectively, all from the same participant. The classification performance of the trained network was subsequently tested on the dataset from P1 that contained 30 videos per alphabet, previously unseen by the trained network.

To evaluate the network’s performance with external participants, it was tested on the dataset from P2, which included 30 videos. This dataset had not been used in the initial training phase. A decline in performance metrics would indicate the presence of unique or additional features in P2. To enhance network applicability, the original training set was expanded by incorporating an additional 50 × 4 videos from P2. The augmented network was then tested on both datasets. This approach is anticipated to significantly improve performance on the second dataset while maintaining high accuracy on the first.

### Schlieren optical system

**Fig. 1:**
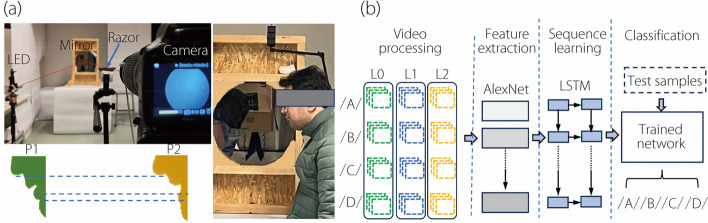
Experimental setup and network model: (**a**) Schlieren optical system and method for recording speech flows, and (**b**) multi-level training and testing of the hybrid CNN-LSTM network for video classification of phonetic alphabet speech flows, i.e., /A/, /B/, /C/, and /D/

The schlieren optical imaging (SOI) system consists of four components: a concave mirror, a point light source, a razor blade, and a video camera for collecting images (Fig. 1a). The mirror, an AD015 telescope mirror (Agena AstroProduct, Cerritos, CA) with a 406 mm diameter, a 1.8 m focal length, and a 45 mm thickness, reflects light from the light-emitting diode (LED) light source, which is covered with a pin-sized hole and placed 3.6 m from the mirror (twice the focal length). The light reflects from the mirror back into the test area and encounters the razor blade, which obstructs approximately half of the light, allowing the remaining half to reach the camera (Canon EOS Rebel T7) and produce an image. Dimming the light enables the camera to better focus on the test participant, who breathes into the test area (right panel, Fig. 1a). Optimal image contrast is achieved by optimizing the location of the light source and the portion of light obstructed by the razor blade. Note that there is no definitive method to measure precisely the amount of light passing from the razor blade to the camera; however, the position of the LED point light source and the razor blade ensures that part of the light is on the blade and the other part hits the camera lens. Obtaining optimal images requires making the point light source very small, complicating the assurance that 50% of the light is on the razor blade and 50% is reaching the camera.

### Acoustic analyses

Acoustic analyses were conducted in MATLAB using both Fourier transform and continuous wavelet transform (CWT). In CWT, various wavelets, including the Morlet wavelet, were used to refine and alter the signal *f(t)*:

1 $$wt(a,b) = \frac{1}{\sqrt{a}} \int\nolimits^{\infty}_{-\infty} f(t) \varPsi \left(\frac{t-b}{a}\right) dt, \quad a > 0$$

*a* is the scaling factor, *b* is the time lag, and $\psi (t)$ is the Morlet wavelet [43]. The wavelet coefficient *wt* represents the degree of similarity between the signal and the wavelet at a particular *a* and *b*. Adjusting *a* and* b* allows for the isolation of spatial and temporal anomalies, such as abrupt changes in patterns. A smaller value of *a* results in a more condensed wavelet and higher frequency, accentuating rapid and sharp fluctuations. Modifying the time lag, *b*, shifts the starting point of the wavelet either forward or backward. The scalogram, which visualizes the time–frequency energy distribution, was derived using the CWT [44].

### CNN-LSTM video classification

A hybrid model combining a CNN with a LSTM network leverages spatial feature extraction with sequential data processing. The model starts with a CNN inspired by AlexNet, known for its effectiveness in image recognition tasks [45] featuring five convolutional layers followed by rectified linear unit activations. Max-pooling layers follow the first, second, and fifth convolutional layers to reduce dimensionality and achieve translational invariance.

The features extracted by the CNN are then input into a bidirectional LSTM (BiLSTM) network to capture temporal dependencies in the data (Fig. 1b). The BiLSTM layer processes sequence information in both forward and backward directions [46], essential for understanding context and progression in temporal data, like video frames or time-series sensor data. The final output goes through a softmax layer (or another suitable activation function) to categorize the video into one of four classifications: /A/, /B/, /C/, or /D /.

Various metrics, derived from the confusion matrix, evaluate the network’s classification performance, including overall accuracy and category-specific measures like precision, sensitivity, specificity, F1 score, receiver operating characteristic (ROC) curve, and area under the curve (AUC). In this four-class system, category-specific metrics adapt from their binary forms using the One-*vs*-Rest approach. The CNN-LSTM model was trained and tested on an AMD Ryzen 3960X 24-Core workstation with 3.79 GHz processors, 256 GB of RAM, and a 24 GB GeForce RTX 3090 GPU (NVIDIA).

## Results

### Schlieren-recorded speech flows

#### Time series of speech flows when pronouncing /A/

**Fig. 2:**
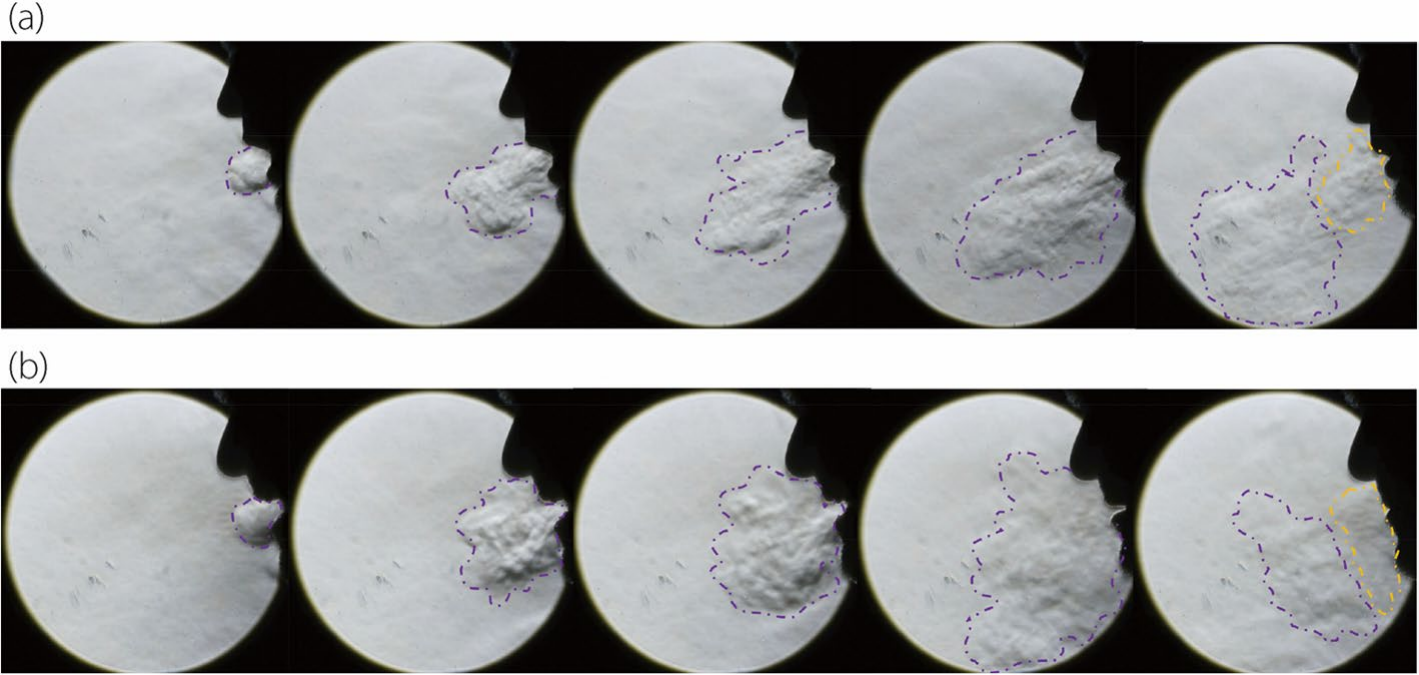
Time series of speech flows captured by schlieren imaging during the pronunciation of /A/ by two participants: (**a**) P1, and (**b**) P2

Figure 2 presents a time series of images illustrating the flow dynamics observed in two participants while pronouncing the letter /A/. Additional details are available in supplementary video S1. During the initial phase of /A/ articulation, the mouth partially opens, leading to the formation of a jet flow at the lips. This jet flow, lasting from 0.2 s to 0.4 s, extends 20–25 cm into the surrounding air at a downward angle. Upon completion of the articulation, the flow from the mouth ceases (indicated in purple), and a secondary flow (indicated in orange) emerges from the nostrils, likely due to the uvula closing the nasopharynx during the articulation of /A/ and the resultant pressure build-up in the nasal cavity.

Comparing different participants, both similarities and discrepancies in /A/ articulation are evident, reflecting alphabet-specific phonetic articulatory features as well as significant diversity even when articulating the same letter. This observation persists despite diligent efforts to ensure consistency in recording, as described in schlieren optical system section. Here, the jet flow from P1 extends a longer distance, while in P2, the flow shows a higher level of dispersion, and the mouth opens wider. Additionally, upon the completion of articulation, P2 completely closes the mouth, while P1 keeps the mouth slightly open, resulting in a softer tone.

#### Speech flows pronouncing /B/, /C/, /D/

**Fig. 3:**
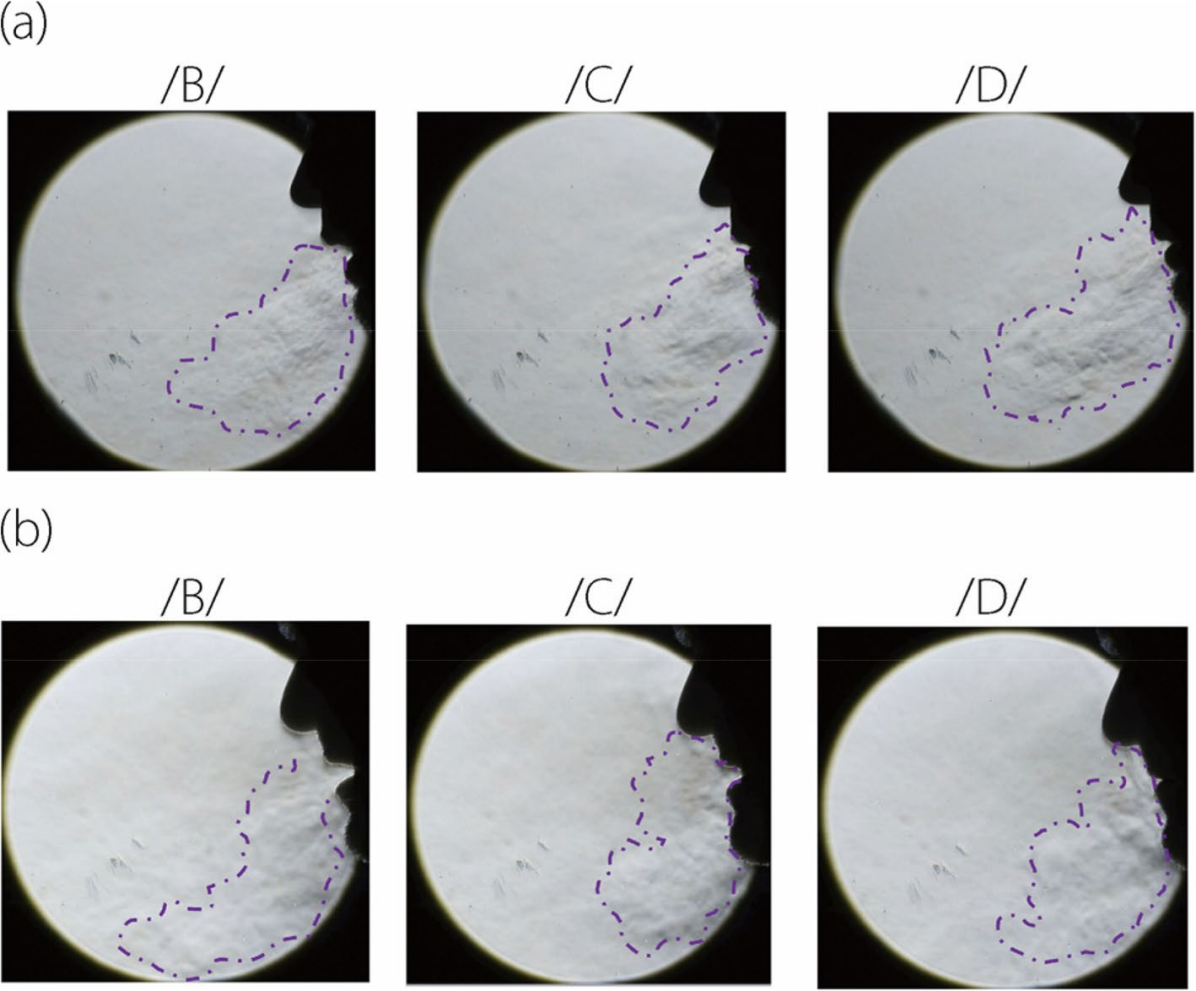
Speech flows captured by schlieren imaging when pronouncing /B/, /C/, /D/ by two participants: (**a**) P1, and (**b**) P2

Distinct differences in speech flow dynamics are observed between /A/ and the other three letters, i.e., /B/, /C/, and /D/, as shown in Fig. 3 and in the supplementary video S1. The three letters also display distinctions in both manners of articulation and the airflow jets accompanying speech. A notable difference is the penetration depth of the jet flows, with /D/ articulation resulting in the shortest penetration, while /A/ has the longest among the four. Such distinctions become more apparent upon viewing the supplementary video S1.

Furthermore, the orientation of the speech flow jets varies, with /A/ forming a 45° angle from the vertical direction and /B/ and /C/ forming a much sharper angle (30°–35°). This variance stems from the relative position between the teeth and lips during pronunciation.

Another distinction is seen in the evolution of the flows and the associated kinematics of the lips and chin producing the flows. From the supplementary video S1, the motions of the lips are clearly observable, with /A/ differing significantly from /B/, /C/, /D/; /A/ involves a wider and longer mouth opening than the others. Subtle differences also exist among /B/, /C/, and /D/, with /B/ featuring an abrupt mouth opening, /C/ showing a retraction of the lower lip and chin, and /D/ having a relatively stable lip/chin position.

The last, yet not the least, difference lies in the flow and articulation manner between different participants. These significant disparities between the two participants, as shown in both Figs. 2 and 3, can result from many factors, such as accent and habit. However, such differences also pose a challenge in identifying the hallmark features that constitute the intelligibility of each letter, as well as in developing a generic AI-based speech reading model based on articulatory flows.

### Sound acoustics, scalogram, and Fast Fourier Transform

#### Pronunciation of /A/

**Fig. 4:**
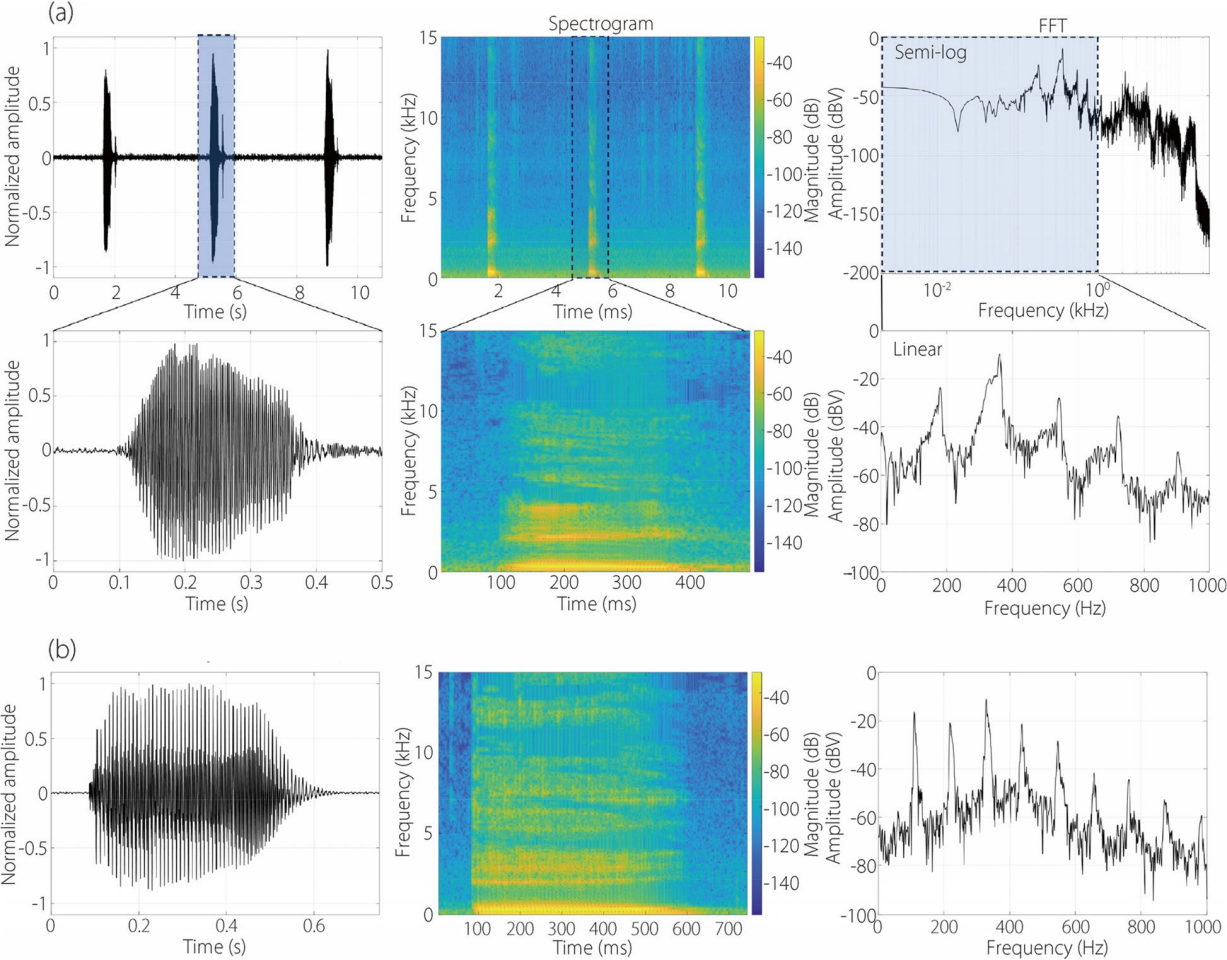
Pronunciation of /A/ and acoustics analyses: (**a**) P1, and (**b**) P2

The soundtrack of /A/ pronunciations, along with their analyses by two participants, is shown in Fig. 4. For a given participant, the pronunciations of the same letter are consistent, as illustrated by the similarity among the three recordings in the first panel in Fig. 4a. Similarities are also observed in the spectrogram, which displays the energy distribution as a function of time and frequency (upper middle panel in Fig. 4a). The lower panels in Fig. 4a show the zoomed soundtrack and spectrogram of the second /A/ pronunciation, lasting around 0.3 s. Several horizontal strips are noted in the spectrogram, whose energy intensity decreases progressively with increasing frequencies, denoting the participant’s fundamental frequency and formants. The two right panels in Fig. 4a depict the Fast Fourier Transform (FFT) of the second /A/ pronunciation in both semi-logarithmic (upper) and linear plots, the latter exhibiting five peaks in the frequency range of 0–1000 Hz.

Both similarities and discrepancies in /A/ pronunciations exist between the two participants in terms of the soundtrack, spectrogram, and FFT profile (Fig. 4b *vs* 4a). The two recordings are similar in their overall patterns, both exhibiting a spindle shape. Also, horizontal stripes are observed in the spectrogram of the P2. However, differences are also apparent. Firstly, the /A/ pronunciation by the P2 lasts longer, i.e., 0.5 s *vs* 0.3 s by the P1. Secondly, the soundtrack patterns between the two participants are slightly different, with the P2’s pattern being more regular than that of the first. Thirdly, the spectrogram of the P2 is less distinctive, indicating a less articulated sound. Lastly, the P2’s FFT profile contains more peaks (9) than that of the P1 (5 peaks). These differences will influence the training process and subsequent performance of the video classification network to be developed.

#### Pronunciation of /B/, /C/, /D/

**Fig. 5:**
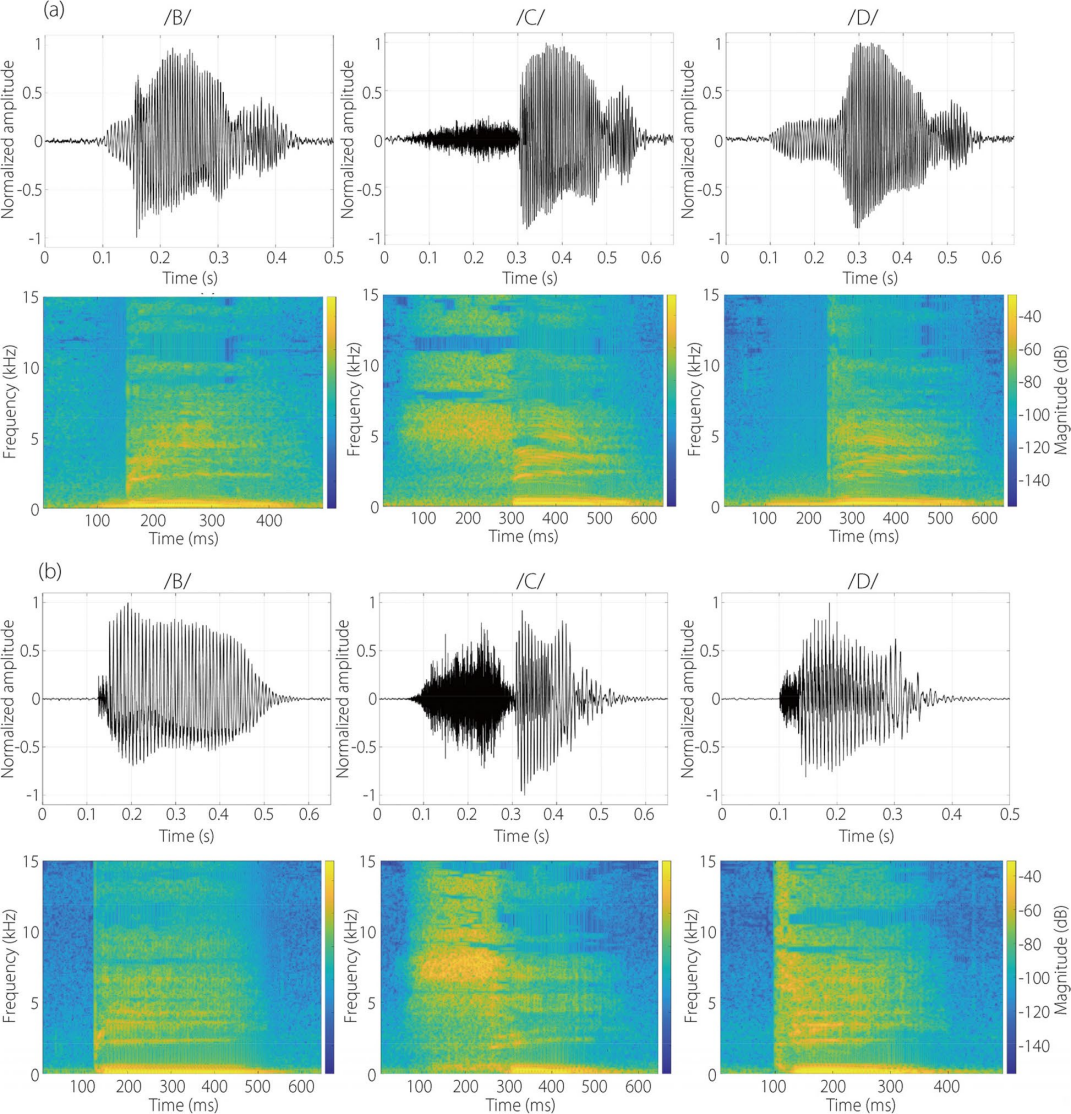
Comparison of the sound recordings and spectrograms for the pronunciation of /B/, /C/, and /D/: (a) P1, and (b) P2

Figure 5 displays the sound recordings and spectrograms for the articulation of the alphabets /B/, /C/, and /D/. For a given participant, each alphabet exhibits a distinctive pattern in the soundtrack and spectrogram. An abrupt shift in energy intensity is observed in the articulation of /B/, /C/, /D/, which is absent in /A/ (Fig. 5*vs* Fig. 4). This shift is most noticeable in /C/, occurring approximately in the middle of the articulation in both participants. The spectrograms differ among the alphabets, each being unique in their energy distribution *vs* time and frequency. Again, both similarities and discrepancies are observed between the two participants for each alphabet, illustrating the individual phonetic characteristics that contribute to intelligible articulation and the signature acoustic qualities of each person.

### CNN-LSTM video classification

#### Network accuracy *vs* number of training videos

**Fig. 6:**
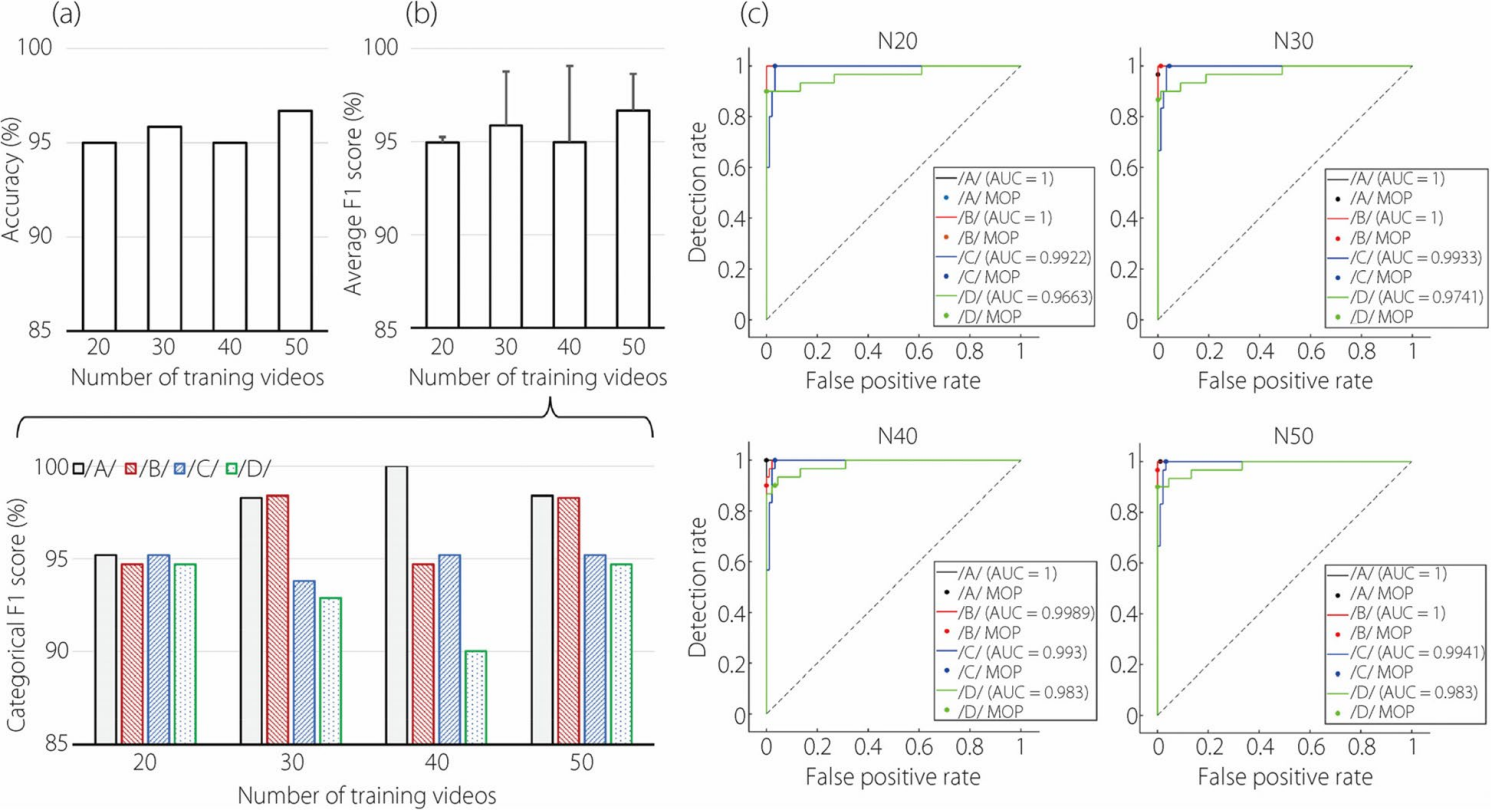
Effect of the number of training videos from P1 on network performance: (**a**) accuracy, (**b**) F1 score (average and category-wise), and (**c**) ROC curves

Figure 6 aims to determine the minimal number of speech flow videos required for effective network training. To this end, four training datasets comprising 80, 120, 160, 200 videos (i.e., 20, 30, 40, and 50 videos per letter, respectively) were utilized to train the CNN-LSTM network. This network was then tested on a distinct dataset containing 120 video clips (30 per letter) from the same participant. For ease of reference, the four trained networks are labeled as N20, N30, N40, and N50, respectively.

In Fig. 6a, it is observed that all four networks achieved a classification accuracy of 95% or higher. This indicates that as few as 20 video clips are sufficient to distinguish the four alphabets. Note that each video clip lasts 1 s and contains 60 frames, which theoretically provides 60 spatial features and 59 time-sequence features. The performance of the network varies among the alphabets, as reflected in the F1 scores shown in Fig. 6b. These scores, both average and category-specific, are above 95%, indicating robust overall performance. Categorical F1 scores exceed 90% for all letters, with /A/ achieving the highest and /D/ the lowest. The ROC curves are similar across the networks, but /D/ consistently demonstrates the poorest performance. Among all trained networks, N50 demonstrates the best performance, albeit by a narrow margin.

#### Network performance on speech flows from a different participant

**Fig. 7:**
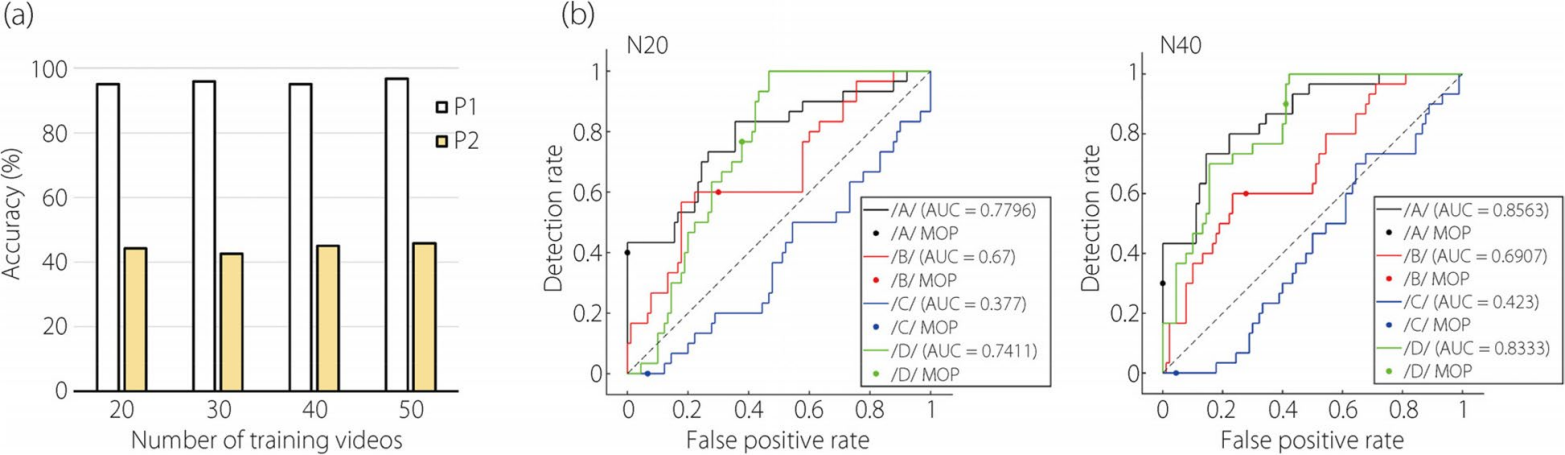
Network performance when tested on a different participant (P2): (**a**) accuracy, and (**b**) ROC curves

The four networks, trained on the P1 dataset, were further tested on an external dataset comprising 120 video clips (30 for each letter) from the P2, resulting in significantly lower accuracies (43%–46%), as shown in Fig. 7a. This decrease indicates the presence of distinct features in P2’s speech flows that are not captured in P1.

Figure 7b displays the ROC curves for N20 and N40 when tested on the P2 test dataset. The letter /C/ is observed to be close to the diagonal line, reflecting the low similarity between the two participants when pronouncing the letter /C/. It is, therefore, necessary to retrain the network by incorporating speech videos from P2 to achieve a network that performs well for both participants.

#### Second round of network training and testing

**Fig. 8:**
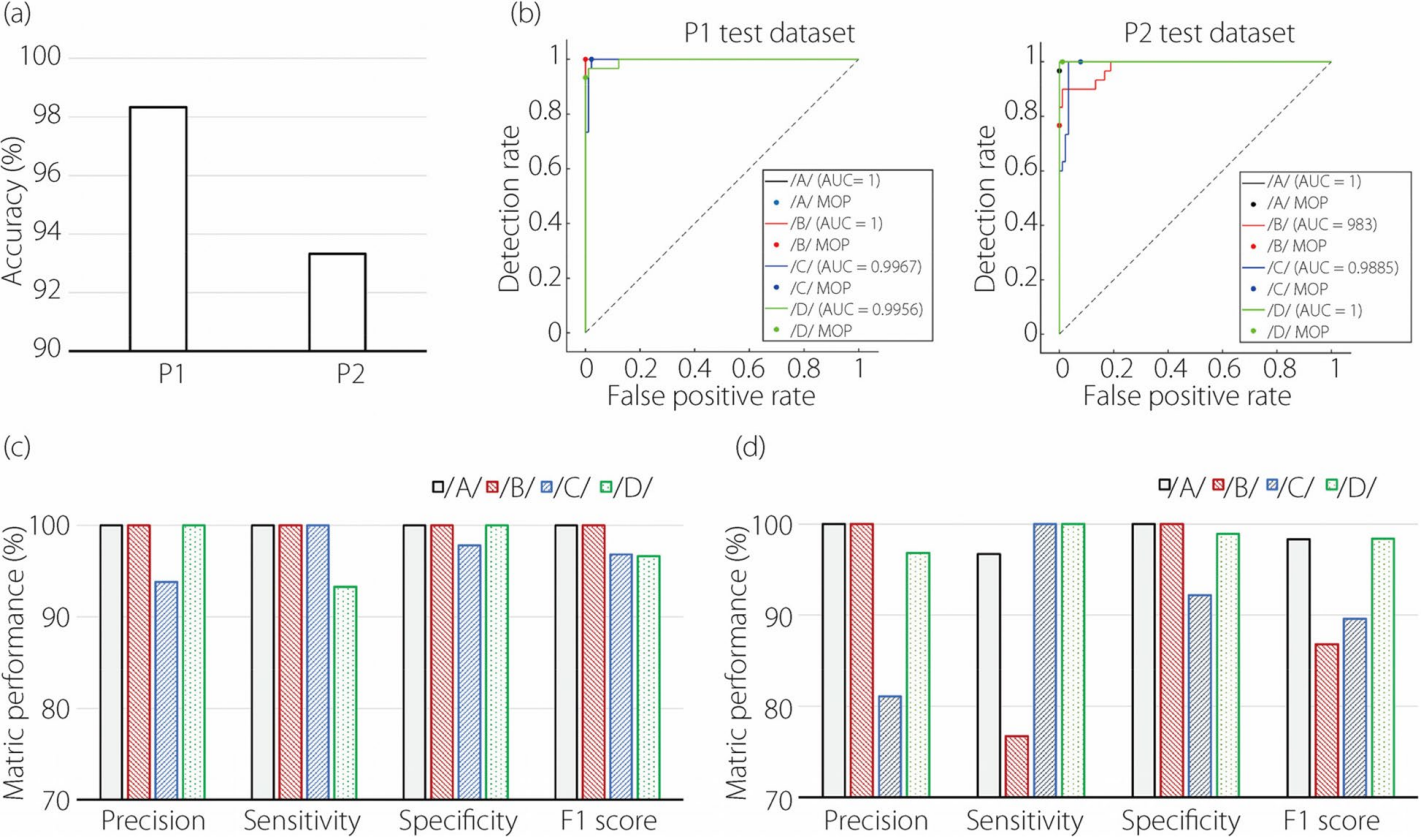
Classification performance of the CNN-LSTM network retrained with 400 video clips (50 per letter per participant) from both participants: (**a**) accuracy on the two test datasets: P1 and P2, each with 30 video clips per letter, (**b**) ROC curves on the two test datasets: P1 and P2, and (**c**) categorical performance on P1, and (**d**) categorical performance on P2

The performance of the retrained model, based on a 400-video dataset (50 per letter per participant) from both participants, is shown in Fig. 8. The retrained network was tested on two datasets, P1 and P2, each containing 120 videos (30 videos per letter). As anticipated, the classification accuracy on P1 remains high at 98.3%, and on P2, it drastically improves from 46% to 93.3% (Fig. 8a). The ROC curves for all four alphabets are close to the upper-left corner, indicating high performance by the retrained network. Figure 8b also shows the network performs slightly less well on P2 than on P1. Additionally, the lowest network performance on P1 is for the letter /D/, while on P2, it is for the letter /B/.

The categorical metric performances, including precision, sensitivity, specificity, and F1 score, are further shown in Fig. 8c (P1) and Fig. 8d (P2). For P1 (Fig. 8c), the two alphabets /C/ and /D/ are more frequently confused, while for P2 (Fig. 8d), the often-confused pair is /B/ and /C/. Moreover, the confusion between /B/ and /C/ for P2 is significantly more frequent than any other pairs (Figs. 8d *vs* 8c). The notably lower values in precision, sensitivity, and F1 score for /B/ in P2 warrant further investigation.

### Misclassification analyses on /B/ pronunciations in P2 test dataset

**Fig. 9:**
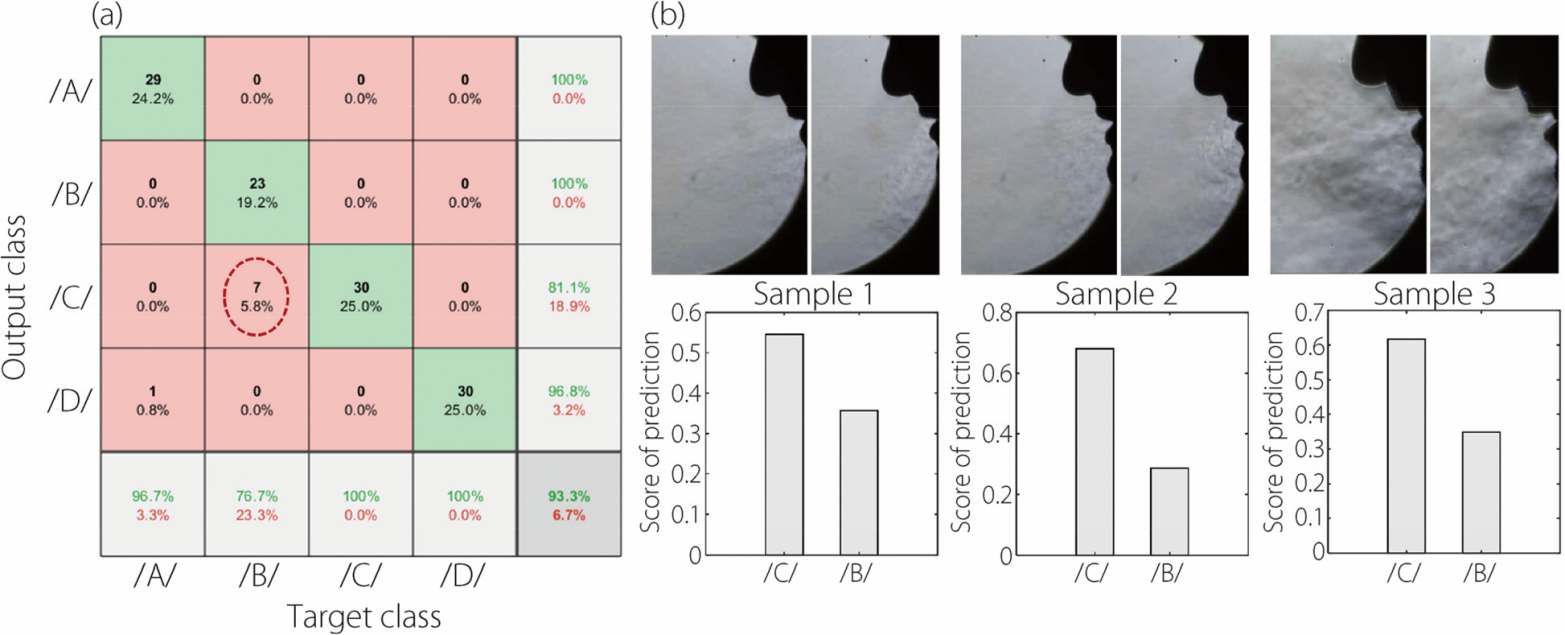
Misclassification analyses of letter /B/: (**a**) the confusion matrix of the retrained CNN-LSTM network when tested on the P2 dataset, (**b**) samples of misclassified videos and their respective prediction scores

The confusion matrix for the retrained CNN-LSTM network tested on the P2 dataset is shown in Fig. 9a. The highest misclassification occurred for the letter /B/, with seven out of thirty /B/ pronunciations misclassified as /C/ (red dotted ellipse, Fig. 9a). All misclassified videos were individually inspected to understand the reasons behind the misclassifications.

Figure 9b presents three typical examples. The articulation of /B/ involves the lips first closing and then quickly opening, whereas during the articulation of /C/, the lips remain open, and the tongue tip initially presses against the teeth, then suddenly retracts, generating specific speech flow patterns. Thus, two snapshots of /B/ speech flows are shown in Fig. 9b, one at the moment of mouth opening and the other at the end of mouth opening. Two anomalies in the first sample in Fig. 9b might contribute to its misclassification: the disproportionally smaller head size relative to the mirror and the low contrast between the flow and background, obscuring the discriminatory features.

The flow patterns of the second sample resemble those of /C/ in Fig. 3, especially at the end of mouth opening. This is consistent with the higher prediction score for /C/ than /B/, as shown in Fig. 9b. In the third sample, the head is disproportionately larger relative to the mirror, and excessive buoyancy plumes introduce nonrelevant features, diluting the alphabet-specific features’ impact. For all three samples, the prediction scores for /C/ are consistently higher than those for /B/ (Fig. 9c), though the superiority is not overwhelming, indicating a close resemblance in the flow patterns associated with these two letters when pronounced by P2.

## Discussion

This study revealed notable distinctions in the spatiotemporal patterns of speech flows among /A/, /B/, /C/, and /D/. The proposed CNN-LSTM network exhibited varying performances on different letters and participants, highlighting both challenges in developing universal speech recognition models and opportunities for improved therapy for patients with speech disorders, as detailed below.

### CNN-LSTM performance *vs* training/testing video sets

In the first classification task, the effect of the number of training videos on model performance was evaluated. The model achieved a classification accuracy of 95% and above for all training sets with 20 videos or more per alphabet (Fig. 6a). Accuracy showed a slight but progressive improvement with an increased number of training videos, reaching the highest accuracy of 96.7% in N50, emphasizing the benefits of a larger training dataset. Each video lasted 1 s and consisted of 60 frames.

Considering alphabet-specific metrics, /A/ had the highest F1 score (Fig. 6), consistent with /A/ being a vowel and /B/, /C/, and /D/ consonants. However, the frequent misclassification of /B/ as /C/ in participant 2 (Fig. 9) indicates a notable similarity in articulatory airflow dynamics between these two letters (Fig. 3), despite their distinct acoustic representations (Fig. 5). The variability in alphabet-specific (categorical) performance among N20–N50 in Fig. 6 suggests that certain alphabetic articulations may require more focused training or present inherent challenges not addressed merely by increasing dataset size. This insight is crucial for further model refinement, especially in optimizing alphabet-specific performance.

Very low performance (44%, Fig. 7) was observed when the model was tested on articulatory flow videos from a different participant. This indicates that while the model performs well in a controlled environment (P1), its ability to generalize and maintain accuracy under more complex scenarios (P2) is limited. This finding is crucial for future model development, emphasizing the need to enhance the model’s adaptability and robustness in varied testing conditions.

The model’s performance significantly improved after retraining with videos from both participants, from 44% to 93% (Fig. 8), underscoring the high inter-participant variability in articulatory flow dynamics and the necessity of including training sets from all relevant sources. Large discrepancies were observed in alphabet-specific metrics, suggesting that while the model excels for certain letters, its ability to generalize across a wider spectrum of alphabets requires further investigation. Moreover, analyses of misclassified samples in Fig. 9 showed that the low quality of certain flow videos contributed to their misclassification; therefore, a standardized video acquisition method is needed to ensure consistency and minimize confounding factors when using the schlieren imaging technique.

It is observed that both the face silhouette kinematics and exhaled flows are features unique to each letter, which can be utilized to differentiate alphabet pronunciations using CNN-LSTM-based video classification. Devergie et al. [47] explored how visual lip gestures can enhance speech intelligibility in scenarios with background noise and multiple speakers. Additionally, it is noted that the human brain is capable of interpreting silent lip movements and translating them into an understanding of speech [48].

### Audio-visual disparities among /A/, /B/, /C/, and /D/

Establishing a one-to-one relationship between alphabetic articulation and speech flows presents challenges. However, it is feasible to infer oral-motor controls from aerodynamic data. In this study, /A/ produced a jet flow with a downward angle of 45° while /B/, /C/, and /D/ produced a much sharper angle (30°–35°). From a phonetic perspective, this difference is attributed to /A/ being a vowel and /B/, /C/, and /D/ being consonants. From an oral-motor control perspective, this difference aligns with the relative motions of the lips, tongue, and jaw during articulation. As depicted in Fig. 2 and supplementary video S1, articulating /A/ involves lowering and then raising the jaw, with the lips open and the tongue resting on the mouth floor. The relatively large oral cavity allows the jet flow to exit the mouth obstructively at an angle of approximately 45° and at a large volume. Furthermore, a larger movement of the jaw accentuates the /A/ sound by releasing more flow. By contrast, the mouth opening is smaller when articulating /B/, /C/, /D/, leading to smaller flow volumes. The jet flows are also influenced by the alphabet-specific oral-motor controls, such as the closing-opening lips in /B/, the teeth touching in /C/, and the tip of the tongue pushing against the alveolar ridge in /D/. Inversely, based on the oral flow dynamics, one can infer the inner oral-motor motions, such as lip opening, tongue-tip retraction, tongue-root advancement, jaw dropping, and larynx lowering, which can further vary in duration, amplitude, and sequence [49]. It is observed that schlieren imaging captures only the side view of the lip and jaw motions, not the movements of the teeth and tongue inside the oral cavity.

Additionally, more nasal leaks in /A/ and /D/ than in /B/ and /C/ were observed in the supplementary video S1. This observation aligns with the findings by Solé [27] that a greater velopharyngeal closure force, leading to a higher pressure build-up in the nasal cavity, results in a larger nasal leak flow. Thus, one can infer the soft palate movements based on the variations in nasal flow.

### Implications for speech therapy

Oral-motor assessments often serve as a starting point in speech therapy and can be repeated to track progress and adjust therapy goals [50]. These assessments evaluate the strength, coordination, and movement of oral components such as the lips, jaw, tongue, and velum [51]. The oral-motor assessment is crucial in identifying the underlying causes of speech and swallowing difficulties. For instance, weak tongue muscles often lead to unclear articulation, and a poor lip closure can result in drooling and challenges with certain speech sounds [52]. Based on the findings of the oral-motor assessment, a speech therapist can develop a tailored therapy plan, which may include exercises to strengthen muscles, improve coordination, and enhance sensory responses.

This study, along with similar future studies based on speech flow visualizations, can offer new insights into articulatory phonetics and provide novel tools for assisting in the diagnosis and therapy of children or adults with speech disorders. Speech production involves a complex feedback mechanism that includes hearing, perception, and information processing within the brain. Similarly, speech therapy often utilizes auditory feedback and lip reading to evaluate and treat speech and language disorders [5, 6]. In contrast to these conventional techniques, this study suggests that a schlieren-based speech flow visualization system can link the three most relevant observables: sound, oral motor, and speech flow. The feedback on therapeutic efficacy is real-time and can be quantitatively measured by a pattern-matching method, such as the degree of sound, lip kinematics, or flow matching their respective normal patterns. Additionally, when synergized with machine learning, this method can devise optimal, patient-specific therapy to correct speech disorders. It is noted that while several studies have used schlieren imaging to study speech flows [26, 29, 30], none have applied it to speech therapy, focusing instead on the flow intensity of nasal/oral vowels in different languages (German [26], Polish [29], and French [30]). Harvey et al. [31] attempted to recover physical audio signals from high-speed schlieren images, but this approach may be limited to shockwaves generated by events such as clapping hands, snapping belts, and cracking towels, where high-frequency signals dominate. It may not be practical for recovering low-frequency speech signals.

### Limitations and future studies

The study could be improved in several aspects. Firstly, including more participants would allow for the evaluation of inter-participant variability in speech flows, thus facilitating the identification of signature phonetic features for each alphabet. Secondly, more alphabets can be considered. However, considering more alphabets also presents an increasing challenge due to the need for more discriminatory features, the introduction of more confounding factors, and the similarity of some letters that may be too subtle to manifest in articulatory flows. Speech exhibits remarkable flexibility and diversity, with subtle and striking differences in pronunciations across regional dialects and accents, such as the noticeable differences between American and British pronunciations. Novel approaches are needed to identify flow-alphabet associations with refined granularity. One promising method is the Meta-Path-Based feature learning method proposed by Zhao et al. [53], which derived hidden features underpinning drug side effects by capturing the heterogeneous associations in meta-paths in an explainable manner. Thirdly, the study only utilized AlexNet to extract spatial features of speech flow morphology. Ablation experiments to evaluate the contributions of individual components to the system’s performance, or comparative experiments with other models, were not performed [54]. Selecting the most appropriate CNN for this task necessitates exploring and evaluating more contemporary methods, including models with more sophisticated and deeper architectures, such as ResNet50 [55] and VGG19 [56], known for their advanced features, and alternatives like MobileNet [57] and EfficientNet [58], known for their simpler structures and greater time efficiency. Each approach offers unique benefits and trade-offs, warranting consideration for optimal performance in this task.

## Conclusions

This pilot study explored the feasibility of speech recognition using schlieren-based articulatory flows of four letters from two participants. The schlieren optical system successfully captured the expiratory flow dynamics of articulatory phonetics, which are beyond human visual capability. Considering the first four English alphabets /A/, /B/, /C/, and /D/, each letter exhibited a unique pattern in flow topology and temporal evolution. However, significant inter-participant variability in flow patterns was also observed between the two participants for each letter. The classification accuracy of the CNN-LSTM network was 95% and above when trained and tested on videos from one participant but dropped to around 44% when tested on a different participant. The network was retrained with videos from both participants, leading to a classification accuracy of 98% on P1 and 93% on P2. Misclassification analysis revealed that low video quality was a contributing factor, underscoring the need for a standardized protocol in video acquisition when utilizing the schlieren imaging technique. Future studies should include more alphabets and participants to enhance the understanding of the flow signatures of articulatory phonetics and develop flow-based speech recognition models.

### Supplementary Information

**Additional file 1: Supplementary video S1. **Schlieren speech flows of /A/, /B/, /C/, /D/, by two participants. 

## Data Availability

The data presented in this study are available upon request from the corresponding author.

## Abbreviations

CNN: Convolutional neural network
LSTM: Long short-term memory
SOI: Schlieren optical imaging
LED: Light-emitting diode
CWT: Continuous wavelet transform
BiLSTM: Bidirectional long short-term memory
ROC: Receiver operating characteristic
AUC: Area under the curve
P1: First participant
P2: Second participant
AI: Artificial intelligence
FFT: Fast Fourier Transform
